# Fatigue after acquired brain injury impacts health-related quality of life: an exploratory cohort study

**DOI:** 10.1038/s41598-021-01617-4

**Published:** 2021-11-12

**Authors:** Elisabeth Åkerlund, Katharina S. Sunnerhagen, Hanna C. Persson

**Affiliations:** 1 Rehabilitation Medicine, Department of Clinical Neuroscience, Institute of Neuroscience and Physiology, University of Gothenburg, Per Dubbsgatan 14, fl. 3, 413 45 Gothenburg, Sweden; 2 Clinic of Rehabilitation Medicine, Sahlgrenska University Hospital, Sahlgrenska University Hospital, Gothenburg, Sweden; 3 Department of Psychology and Counselor, Sahlgrenska University Hospital, Sahlgrenska University Hospital, Gothenburg, Sweden

**Keywords:** Fatigue, Cognitive neuroscience, Risk factors, Signs and symptoms, Brain injuries, Stroke, Quality of life

## Abstract

This study aimed to identify the consequences of fatigue, fatigability, cognitive and executive functioning, and emotional state on health-related quality of life (HRQoL) in a clinical group of outpatients after acquired brain injury (ABI). This cross-sectional retrospective study included assessing outpatients at a rehabilitation clinic with WAIS-III working memory and coding subtests, and self-rating scales (Fatigue Impact Scale, Dysexecutive Questionnaire, Hospital Anxiety and Depression Scale, and the dimension of health-related quality of life from EQ-5D-3L). The predictive variables were investigated using a binary logistic regression with HRQoL as the dependent variable. Descriptive statistics and correlations were analyzed. Participants reported a lower than average HRQoL (95%), fatigue (90%), and executive dysfunction (75%). Fatigue had a significant impact and explained 20–33% of the variance in HRQoL with a moderate significance on depression (p = 0.579) and executive dysfunction (p = 0.555). Cognitive and executive function and emotional state showed no association with HRQoL. A lower HRQoL, as well as fatigue and cognitive and executive dysfunctions, are common after ABI, with fatigue is a partial explanation of a lower HRQoL.

## Introduction

Acquired brain injury (ABI) refers to any brain damage after birth that may be caused by a stroke, traumatic brain injury, or other medical problems^1^. Regardless of the cause, ABI survivors are treated in the same rehabilitation units that provide support for one’s functioning, activity, participation, and quality of life^2,3^.

The concept of health-related quality of life (HRQoL) focuses on the subjective experience of wellbeing after injury, based on physical, mental, and social aspects^4^. The term quality of life (QoL) has been previously used more broadly, often including aspects of HRQoL. QoL after ABI was early associated with the severity of injury due to cognitive deficits^5–7^, but with an increasing focus on the individual's biopsychosocial conditions and sociodemographic context. Thus QoL has been seen to be associated with comorbidity^8,9^, participation^3^, mood^2,10^, self-esteem^10^, coping strategies^11^ and acceptance^12^. QoL has been reported to improve^13^, but also deteriorate over time^2,3^.

Cognitive impairments in mental speed, attention, memory, working memory (WM), communication and executive functioning are common after ABI^8,14^. WM is conceptualized as the amount of information an individual can process at a given time to use in performing cognitive activities, mental organization of information and problem-solving, which are some of the cores of the executive functions^15^. Also psychological distress like anxiety, depression and perceived stress are seen as long term reactions after ABI^14^. However, fatigue is often reported to be the subjectively most common disability, estimated in 70–80% of patients after traumatic brain injury^16^ and in 23–77% of patients after stroke^17^, restricting daily life and returning to work^16^. Fatigue is also a common consequence of a broad range of neurological and other diseases^18,19^. It is also identified in neurologically intact persons, with a 24% lifetime prevalence^20^. Several symptoms overlap between fatigue and depression, and since fatigue is a diagnostic criterion for depression^21^, they can easily be misunderstood and mask one another. However, research provides increasing evidence that fatigue can be seen as a primary consequence of ABI^17,22^. Studies suggest the cause to be cerebral biochemical changes^17,23–25^, or a complex interaction of physiological, cognitive and psychological disabilities^26^. There seems to be a limited relationship between fatigue and the severity of ABI or the extent of the remaining disability^8,16,27^. Some studies report associations between fatigue and aspects of cognition such as attention, memory, and executive functions^17^, psychological distress^28–30^, or a lower QoL^31–33^. However, other studies do not report the same^34,35^. Cognitive fatigue, also called mental fatigue, differs from physical, emotional, and stress related fatigue; it is often described as a chronic mental tiredness and a lack of energy, a sense of exhaustion lasting long after a mental activity^36^. Different aspects of cognitive fatigue have been suggested, including *fatigue*—as the subjective experience of weariness, and *fatigability*—an objective perspective about how performance weakens over time, confirmed by results in neuropsychological testing or other activity^19,37^.

In the context of rehabilitation, there is an underlying assumption that exercise and interventions lead to “a better life", an abstract and subjective concept. As the terms of cause and effect of consequences vary in the literature, there is a continuing need to further understand the complexity of consequences after ABI, and how they affect a person’s HRQoL^33^, to find adequate individualized therapeutic interventions as well as generalized guidelines.

This study aimed to identify the consequences of subjective fatigue, objectively measured fatigability, cognitive and executive functioning, and emotional state on HRQoL in a clinical outpatient group after ABI.

## Results

Forty-one participants were included in the study. The majority were diagnosed with stroke, followed by traumatic brain injury and other acquired brain injury such as encephalitis (1 person), neurosarcoidos (1 person) and post planned brain surgery (3 persons). All participants except one, were included 3 months to 2.5 years since injury. One participant was included nine years after the injury. The vast majority of the participants reported low HRQoL, problematic fatigue, executive problems and also performed below average in processing speed. Table 1 shows more details.

**Table 1:** The characteristics of the participants with acquired brain injury (n = 41), background factors, neuropsychological and self-rated assessments.

Background factors			
Sex (male/female)	19/22		
**Diagnosis frequency, n (%)**			
Stroke	31 (76)		
Traumatic brain injury	5 (12)		
Other acquired brain injury	5 (12)		
**Education, years of studies, n (%)**			
1–9 years	4 (10)		
9–12 years	18 (44)		
12 + years	19 (46)		
Age, years, M (SD), Md (min–max)	48.5 (10.2)	51 (22–63)	
**Time since injury, weeks**			
M (SD), Md (min–max*)*	49.0 (77.5)	32 (12–500)	

Neuropsychological assessments	M (SD)	Md (min–max)	% compared to average or cut offs marking problems
Digit Span forwards (raw score), (*n* = 40)	5.4 (1.1)	5 (3–8)	
Digit Span backwards (raw score)	3.9 (1.1)	4 (2–7)	
Block Span forward (raw score)	5.2 (1.1)	5 (2–8)	
Block Span backwards (raw score)	4.7 (1.2)	4 (2–9)	
WMI (index)	87.7 (14.0)	92 (57–106)	80% below average
Coding (scaled score)	6.0 (2.3)	6 (2–13)	92% below average

Self-rated assessments	M (SD)	Md (min–max)	% compared to average or cut offs marking problems
EQ5D index	0.6 (0.3)	0.7 (− 0.2–1)	95% < M = 0.91 37% < M = 0.62
FIS (raw score)	65.7 (40.1)	67 (0–150)	90% above Md = 13.5
DEX (raw score), (*n* = 40)	26.5 (11.8)	27 (3–46)	75% above cut off 19 points
HADS A (raw score)	7.4 (4.8)	6 (0–18)	39% above cut off 8 points
HADS D (raw score)	5.4 (3.7)	5 (0–15)	25% above cut off 8 points

*N* participants, *M* mean, *SD* standard deviation, *Md* median, *min–max* minimum–maximum.

*Assessments:* Digit Span, Block Span, WMI (index of Digit Span, Arithmetics, and Letter Number Sequencing) and Coding from the WAIS-III NI Wechsler Adult Intelligence Scale 3rd edition. *FIS* Fatigue Impact Scale, *DEX* Dysexecutive Questionnaire, *HADS A resp. D* Hospital Anxiety and Depression Scale, *EQ5D index* EuroQoL-5 dimensions (EQ-5D-3L).

The multivariable logistic regression was performed with the dichotomized variable HRQoL as the dependent variable with 26 observations in Group 0 (results above 0.62) and 15 observations in Group 1 (results below 0.62). Independent variables showed no multicollinearity. The stepwise analysis is presented in Table 2.

**Table 2:** Flow chart of 4 steps in the multivariable regression analysis in the participants with acquired brain injury, (n = 40).

	Step 1	Step 2	Step 3	Step 4
Analysis	Cross tabulation of dichotomy variables to exclude variables < 5 observations	Collinearity check between variables to exclude correlations Spearman’s rho > 0.7	Univariate logistic regressions with HRQoL as dependent variable to exclude p < 0.25, Wald test	Multivariable logistic regression—stepwise back and forth to exclude variables not reaching the significance level of p < 0.05
Variables ruled out	–	Working memory	Processing speed, executive function, anxiety, age	Executive functioning, depression and sex
Remaining variables	HRQoL and sex	Fatigue, processing speed, executive function, anxiety, depression, sex, age	Fatigue, executive functioning, depression and sex	Fatigue

*HRQoL* Health Related Quality of Life.

One participant was excluded from the regression analysis due to missing data concerning executive functioning.

Fatigue remained the only variable that contributed statistically significantly to the model in the regression analysis, explaining between 23 and 31% of the variance in HRQoL (OR 1.031, 95% CI 1.009–1.053. *p* = value 0.005, Nagelkerke *R*^2^ 0.312).

HRQoL showed no statistical correlations with any of the included variables. Fatigue had a moderate correlation with executive functioning and depression. Moderate correlation levels were also seen between executive functioning, anxiety, and depression, and between anxiety and depression (Table 3).

**Table 3:** Spearman’s rank order correlation between assessments and background factors in the participants with acquired brain injury (n = 41).

Function	HRQoL	Fatigue	Working memory	Psycho-motor processing	Executive function (n = 40)	Anxiety	Depression
Assessment	EQ5D index	FIS	WMI	Coding	DEX	HADS A	HADS D
FIS	0.411**						
WMI	0.393*	− 0.147					
Coding	0.101	− 0.105	0.421**				
DEX, (n = 40)	− 0.174	0.555**	− 0.067	0.038			
HADS A	− 0.187	0.409**	− 0.164	− 0.017	0.603**		
HADS D	− 0.239	0,579**	0.002	0.035	0.631**	0.699**	
Type of diagnosis	− 0.078	0.484**	0.002	− 0.073	0.444**	0.360*	0.188
Time since injury	− 0.085	0.364*	− 0.271	− 0.208	0.206	0.286	0.236
Sex	− 0.217	0.149	− 0.343*	0.241	0.152	0.102	0.328
Age	0.031	0.025	0.320*	0.166	− 0.047	0.064	0.100

Spearman's rank order correlation (sign. 2-tailed) *p ≤ 0.05, **p ≤ 0.01.

*HRQoL* health-related quality of life, *EQ5D index* EuroQoL-5 dimensions (EQ-5D-3L), *FIS* Fatigue Impact Scale, WMI (index of Digit Span, Arithmetics and Letter Number Sequencing) and Coding from the WAIS-III NI Wechsler Adult Intelligence Scale 3rd edition. *DEX* Dysexecutive Questionnaire, *HADS A resp. D* Hospital Anxiety and Depression Scale, *Diagnosis* stroke/traumatic brain injury/other acquired brain injury.

## Discussion

The results showed how subjective fatigue influenced HRQoL, cognitive and executive functioning in a clinical outpatient group after ABI. The study recognizes fatigue to contribute to the variance of 20 to 33% in the reduced HRQoL, supporting the increased focus in clinic and research of fatigue as an impact on activity and HRQoL^33,38,39^. More than 37% expressed their HRQoL at a level that indicates a functional capacity corresponding to a level below work ability^40^, and almost all participants reported a lower HRQoL than the Swedish average^41^. The lower HRQoL seen in this group of patients after an ABI conforms with earlier findings^5–7,42^, and emphasizes the importance of addressing the causes and interventions to enable a higher HRQoL for those affected.

The problematic fatigue experienced by the majority of the population (90%) is consistent with earlier findings^33,43^ and the increasing attempts to understand the concept of fatigue. Earlier research has highlighted a distinction between self-rated fatigue and fatigability, apparent in objective tests and activities that require processing speed, suggesting different cerebral networks for respectively fatigue and fatigability^44^. In this study no statistical associations or differences were found between self-rated fatigue and the performance in the test measuring processing speed. Even though the group underperformed in both areas they performed overall better in the neuropsychological test compared to how they subjectively reported their functions. It may be likely that the two-minute long effort in the test Coding is too short to fully capture the problem with fatigability. The results could however indicate the suggested difference between fatigue and fatigability, that the neuropsychological test demand high focus, but only for a very short time, and thus seems less energy consuming than the fatigability in an everyday setting described in the self-assessments.

As fatigue permeates everyday commitments in life it could be speculated that the long-lasting problem with fatigue after ABI has become more acknowledged as the medical progress has led to better outcomes with less physical and cognitive deficits. The consequences of fatigue are, thus, given a new and more problematic perspective when a large proportion of patients are confronted with expectations of going back to work and everyday life. For some, fatigue may be overwhelming and a major obstacle to getting back to one’s life. This could explain the low association between fatigue and time since injury. The widespread time since injury for this group shows typical variance for patients of the outpatient clinic, where some individuals have a prolonged need for rehabilitation to achieve strategies to master and overcome fatigue, even long after ABI.

The participants underperformed in cognitive functions of processing speed and working memory aspects, compared to the average population, and a vast majority of the group reported lower executive functioning than average. However, these findings showed no impact or correlation with HRQoL, conforming to other studies^34,35^. As the concept of health-related HRQoL focuses on a broad subjective experience of wellbeing these results suggest that reduced cognitive ability and executive functions may change the ability and functions to troublesome levels, but not per se leads to a lower HRQoL as HRQoL also depend on physical, mental and social aspects. It could also be suggested that a lower cognitive and executive capacity may be experienced as difficulties handling and mentally lasting in activities. The association between fatigue and executive functioning could be explained as executive fatigue^37^—the vulnerability of not mentally enduring everyday demands of planning, organizing, and keeping track.

Previous research indicates that individuals with low executive functioning tend to use more passive coping styles, leading to poor quality of life^45^. The widespread scores within all tested domains reflect how heterogeneous the sample is, where even a mild ABI can cause multiple consequences for some patients, but not for others. The results of this study suggest that fatigue hinders the activity and participation of an individual that is expressed as a lower HRQoL. The constant fatigue risks getting so fundamental that the very idea of not being able to stress oneself in thinking and everyday activities is expressed as a lower HRQoL, but not necessarily emotional distress.

This study showed no association between HRQoL and emotional distress, contrary to earlier findings^2,10^. Only 25% of the participants showed some signs of depression, while symptoms of anxiety were slightly more common, consistent with previous studies^46^. This suggests that ABI does not automatically cause emotional distress—it is dependent on a broader spectrum of consequences. One aspect could be that patients in a rehabilitation program are more hopeful to get help and feel more cared for, and therefore, are more emotionally stable.

The low HRQoL, and the impact of fatigue on HRQoL for the ABI population indicated in the present study strengthens the need to include psychoeducation, treatments, and strategies to manage fatigue in rehabilitation as a tool for patients to engage in social activities, and when possibly, return to work, as this usually indicates good HRQoL. Few studies have focused on HRQoL as the main outcome after ABI, but the current finding of a relationship between fatigue and HRQoL is in line with some previous reports^31–33^. Increased research in the psychosocial perspective is suggested to identify why some ABI patients seem more affected than others. Further research is required to know more about how fatigue and other consequences develop and impact life over time, to ensure a better HRQoL for the person after ABI.

This study has several limitations. The clinical setting included several dropouts and exclusions for a variety of medical reasons. This left a somewhat skewed group in terms of the spread of diagnoses and time since injury. The patient group was initially selected due to their limited working memory (WM). Comparing results from objective and subjective assessments is always a risk, due to individual interpretations. However, these subjective experiences cannot be detected objectively.

In conclusion, this study strengthens the understanding of lower HRQoL, as well as fatigue, cognitive and executive dysfunction as common consequences after ABI. Fatigue may explain between 20 and 33% of the variance in HRQoL, while cognitive and executive functioning, and emotional state showed no statistical correlation with HRQoL in this study. These findings emphasize the importance of addressing HRQoL as an outcome after ABI and to focus on fatigue treatments during the rehabilitation process, to support the fatigued person in finding strategies to cope with activity and participation for a better HRQoL.

## Materials and methods

### Study design and procedure

#### Participants

**Figure 1:**
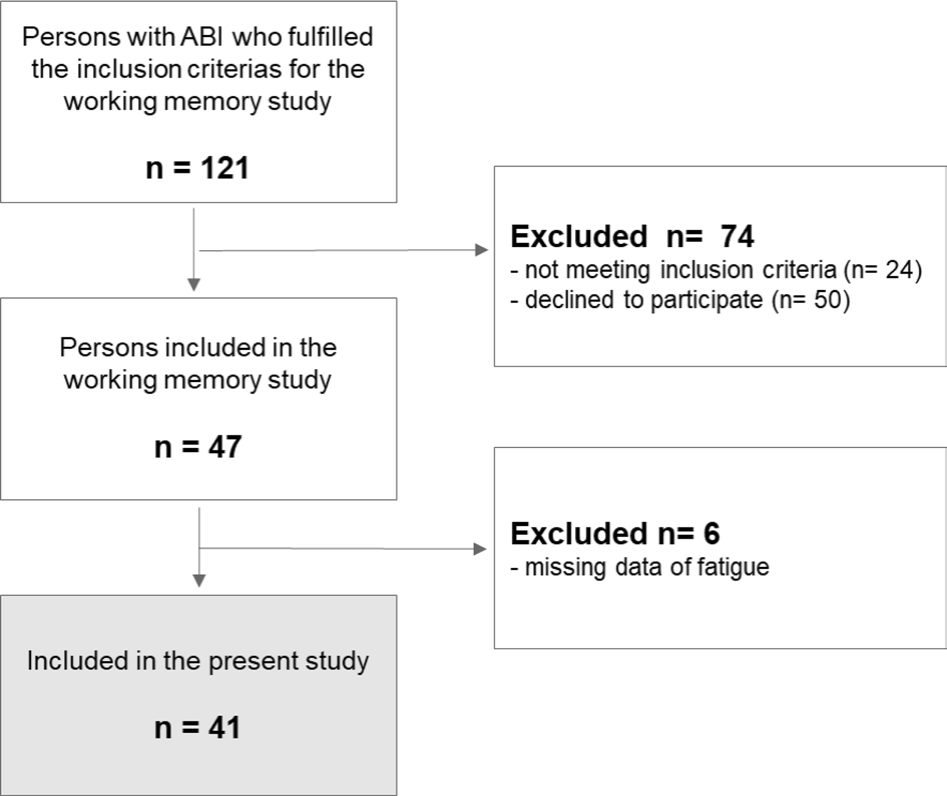
Flowchart of the study participants.

Participants in this cross-sectional study were patients admitted to the Outpatient Neurorehabilitation program at the Clinic of Rehabilitation Medicine, Sahlgrenska University Hospital, Sweden. The clinic offers neurocognitive rehabilitation, in interdisciplinary teams, to patients in the post-acute to chronic phase after ABI. This is based on individualized needs and goals and often focuses on the ability to return to work. The present study is part of a larger project, where all patients had consecutively been assessed within a study of computerized working memory training^47,48^, for which a reduced working memory (WM), either verbal or spatial, was set as inclusion criteria. Reduced WM was determined by a score of _5 digits/blocks forwards and/or_4 digits/blocks reversed on the WAIS III Digit span and WAIS-III-NI Span board^49^, results described as marginal to normal limits by M. Lezak^50^. The exclusion criteria included aphasia, insufficient ability to read or talk Swedish, and/or medical reasons. Participants with a completed assessment of fatigue were also included in the current study (Fig. 1). Individual assessments and self-rating scales were performed during the first two weeks of the rehabilitation period by the neuropsychologist. Demographic data were collected from medical charts. All participants received verbal and written information about the study and their right to renounce from further participation. Participants gave written informed consent prior to the assessment according to the ethical principles of the Declaration of Helsinki.

### Self-rating assessments

The *Fatigue Impact Scale* (FIS)^51^ focuses on the perceived impact of fatigue on cognitive, physical, and psychosocial activities. The questionnaire consists of 40 questions, each rated from 0 (no problem) to 4 (very much a problem). In Sweden, the median score on the FIS is 13.5 (minimum 2–maximum 44)^41^.

*EuroQol EQ-5D-3L* was used to estimate HRQoL by measuring five dimensions: mobility, self-care, usual activities, pain/discomfort, and anxiety/depression. Each dimension is rated on three levels: no problems, some problems, and extreme problems^52^. The reported health states combine into an EQ5Dindex of − 0.594–1.0, where higher scores indicate higher HRQoL. An experience-based value set determined a median of 0.91 for the Swedish population^53^, while another Swedish study proposed a cut-off value at 0.62 to predict the ability to return to work^40^, thus an indication of a higher functional ability.

*The Dysexecutive Questionnaire* (DEX) of the Behavioural Assessment of the Dysexecutive Syndrome^54^ measures experienced dysexecutive problems in emotion, personality, motivation, behavior, and cognition through 20 items scored on a 5-point Likert scale. Higher scores indicate more problems. A score of < 10 indicates optimal functioning, 10–18, sub-optimal functioning within normality, 19–28, moderately dysexecutive functioning and > 28, important degree of dysexecutive disorder with severe pathologies^55^.

Emotional state was evaluated using the *Hospital Anxiety and Depression Scale*^56^. Fourteen questions measure levels of anxiety and depression; each question is scored between zero and three points. For anxiety (HADS A) and depression (HADS D), a score of 8–10 indicates a mild, 11–14 points a moderate, and scores > 15 points indicate a severe condition^57^.

### Neuropsychological assessments

Cognitive functions were assessed with sub-tests from the Swedish version of the *Wechsler Adult Intelligence Scale III* (WAIS-III NI)^58^. Results from the Digit Span and Span Board determined the WM^50,59^. The Digit Span, Letter-Number Sequencing and the Arithmetic subtest form the Working Memory Index (WMI) (M = 100 points; SD = 15). The Coding subtest measures processing speed and fatigability, as the participant matches numbers and symbols according to a key, as fast as possible for 120 s. Scores vary between 1 and 19, with mean = 10 (SD = 3). Higher results correspond to better performance.

### Statistical methods

Descriptive statistics are presented as frequencies and/or percentages, as means (M), standard deviations (SD), medians (Md), and minimum–maximum (min–max) values. The HRQoL was dichotomized into two groups from a cut-off value of 0.62 in the EQ5D™^40^.

A multivariable logistic regression analysis was conducted. The independent variables were working memory index (WMI), processing speed (Coding), executive functioning (DEX), anxiety (HADS A), depression (HADS D), fatigue (FIS), sex, and age. Their impact was studied on HRQoL (EQ5Dindex), which was the dependent variable. Non-parametric Spearman’s rho was used to examine correlations between variables, with *p* values of significance as < 0.26 (little, if any), 0.26–0.49 (low), 0.50–0.69 (moderate), 0.70–0.89 (high), and ≥ 0.90 (very high)^60^.

The multivariable logistic regression analysis was performed in four steps. In the first step, a cross tabulation was used to identify and exclude variables that contained less than five observations in any subgroup. In the second step, collinearity between predictor variables was checked using Spearman’s rank correlation test for ordinal variables. Variables with a correlation above 0.7 were considered for collinearity. In the third step, a series of univariate logistic regressions were performed on all variables not excluded in previous steps to detect significant variables (a significant level of p < 0.25, Wald test). In the fourth step, significant variables were tested in a multivariable logistic regression in a stepwise model in which variables not reaching the significance level of p < 0.05 were excluded. Variables previously excluded in steps 2–4 were reinserted in the final model, one by one, to check for possible significant effects in the model (p < 0.05, Wald test). Models are presented with unstandardized coefficients, p-values, and odds ratios with 95% confidence intervals. The logistic regression models were tested with Nagelkerke R^2^ (the value 1 indicates a perfect level explained by the model) and the Hosmer–Lemeshow test (p > 0.05 indicates good fit). Statistical analyses were performed using the IBM Statistical Package for Social Sciences (SPSS version 24.0, for Windows).

### Ethical considerations

This study was executed within a clinical development project with permission from the head of the department. Ethical considerations were made throughout the study to ensure the participants’ safety and integrity according to professional and hospital ethical rules. The participants provided written informed consent in accordance with the Declaration of Helsinki, and the study was carried out in accordance with the recommendations of the regional ethics committee in Stockholm.

## Data Availability

The datasets used or analyzed during the current study can be made available by the corresponding author upon reasonable request. However, according to the Swedish Secrecy Act 24:8, an interested researcher must first apply and receive approval from the Swedish Ethical Review Authority.

## References

[1] Teasell R (2007). A systematic review of the rehabilitation of moderate to severe acquired brain injuries. Brain Inj..

[2] Goverover Y, Genova H, Smith A, Chiaravalloti N, Lengenfelder J (2017). Changes in activity participation following traumatic brain injury. Neuropsychol. Rehabil..

[3] Verdugo MA, Fernandez M, Gomez LE, Amor AM, Aza A (2019). Predictive factors of quality of life in acquired brain injury. Int. J. Clin. Health Psychol..

[4] Fayers PM, Machin D (2016). Quality of Life: The Assessment, Analysis and Reporting of Patient-Reported Outcomes.

[5] Cumming TB, Brodtmann A, Darby D, Bernhardt J (2014). The importance of cognition to quality of life after stroke. J. Psychosom. Res..

[6] Reddy RP, Rajeswaran J, Devi BI, Kandavel T (2017). Cascade of traumatic brain injury: A correlational study of cognition, postconcussion symptoms, and quality of life. Indian J. Psychol. Med..

[7] Azouvi P (2016). Disability and health-related quality-of-life 4 years after a severe traumatic brain injury: A structural equation modelling analysis. Brain Inj..

[8] van Rijsbergen MWA, Mark RE, Kop WJ, de Kort PLM, Sitskoorn MM (2019). Psychological factors and subjective cognitive complaints after stroke: Beyond depression and anxiety. Neuropsychol. Rehabil..

[9] Chen K, Marsh EB (2018). Chronic post-stroke fatigue: It may no longer be about the stroke itself. Clin. Neurol. Neurosurg..

[10] Lapadatu I, Morris R (2019). The relationship between stroke survivors' perceived identity and mood, self-esteem and quality of life. Neuropsychol. Rehabil..

[11] Boosman H (2017). Predictors of health-related quality of life and participation after brain injury rehabilitation: The role of neuropsychological factors. Neuropsychol. Rehabil..

[12] Van Bost G, Van Damme S, Crombez G (2017). The role of acceptance and values in quality of life in patients with an acquired brain injury: A questionnaire study. PeerJ.

[13] Gould KR, Ponsford JL (2015). A longitudinal examination of positive changes in quality-of-life after traumatic brain injury. Brain Inj..

[14] Ponsford JL (2014). Longitudinal follow-up of patients with traumatic brain injury: Outcome at two, five, and ten years post-injury. J. Neurotrauma.

[15] Kumar S, Rao SL, Chandramouli BA, Pillai S (2013). Reduced contribution of executive functions in impaired working memory performance in mild traumatic brain injury patients. Clin. Neurol. Neurosurg..

[16] Mollayeva T (2014). A systematic review of fatigue in patients with traumatic brain injury: The course, predictors and consequences. Neurosci. Biobehav. Rev..

[17] Hinkle JL (2017). Poststroke fatigue: Emerging evidence and approaches to management: A scientific statement for healthcare professionals from the American Heart Association. Stroke.

[18] Cieza A (2015). PARADISE 24: A measure to assess the impact of brain disorders on people's lives. PLoS One.

[19] Kluger BM, Krupp LB, Enoka RM (2013). Fatigue and fatigability in neurologic illnesses: Proposal for a unified taxonomy. Neurology.

[20] Walker EA, Katon WJ, Jemelka RP (1993). Psychiatric disorders and medical care utilization among people in the general population who report fatigue. J. Gen. Intern. Med..

[21] American Psychiatric Association. *Diagnostic and statistical manual of mental disorders* 5th edn, (Washington, DC, 2013).

[22] Schönberger M, Herrberg M, Ponsford J (2014). Fatigue as a cause, not a consequence of depression and daytime sleepiness: A cross-lagged analysis. J. Head Trauma Rehabil..

[23] Becker K (2015). Poststroke fatigue: Hints to a biological mechanism. J. Stroke Cerebrovasc. Dis..

[24] Dobryakova E, Genova HM, DeLuca J, Wylie GR (2015). The dopamine imbalance hypothesis of fatigue in multiple sclerosis and other neurological disorders. Front. Neurol..

[25] Wylie GR (2017). Cognitive fatigue in individuals with traumatic brain injury is associated with caudate activation. Sci. Rep..

[26] Zgaljardic DJ (2014). Neuropsychological and physiological correlates of fatigue following traumatic brain injury. Brain Inj..

[27] Wylie GR, Flashman LA (2017). Understanding the interplay between mild traumatic brain injury and cognitive fatigue: Models and treatments. Concussion.

[28] Galligan NG, Hevey D, Coen RF, Harbison JA (2016). Clarifying the associations between anxiety, depression and fatigue following stroke. J. Health Psychol..

[29] Wu S, Barugh A, Macleod M, Mead G (2014). Psychological associations of poststroke fatigue: A systematic review and meta-analysis. Stroke.

[30] Chen YK (2014). Poststroke fatigue: Risk factors and its effect on functional status and health-related quality of life. Int. J. Stroke.

[31] Cantor JB (2008). Fatigue after traumatic brain injury and its impact on participation and quality of life. J. Head Trauma Rehabil..

[32] Pollock A, St George B, Fenton M, Firkins L (2014). Top 10 research priorities relating to life after stroke—Consensus from stroke survivors, caregivers, and health professionals. Int. J. Stroke.

[33] van Markus-Doornbosch F, van der Holst M, de Kloet AJ, Vliet Vlieland TPM, Meesters JJL (2020). Fatigue, participation and quality of life in adolescents and young adults with acquired brain injury in an outpatient rehabilitation cohort. Dev. Neurorehabil..

[34] Chen CH, Hung KS, Chung YC, Yeh ML (2019). Mind-body interactive qigong improves physical and mental aspects of quality of life in inpatients with stroke: A randomized control study. Eur. J. Cardiovasc. Nurs..

[35] Esbjornsson E, Skoglund T, Sunnerhagen KS (2013). Fatigue, psychosocial adaptation and quality of life one year after traumatic brain injury and suspected traumatic axonal injury; evaluations of patients and relatives: A pilot study. J. Rehabil. Med..

[36] Staub F, Bogousslavsky J (2001). Fatigue after stroke: A major but neglected issue. Cerebrovasc. Dis..

[37] Moller MC, Nygren de Boussard C, Oldenburg C, Bartfai A (2014). An investigation of attention, executive, and psychomotor aspects of cognitive fatigability. J. Clin. Exp. Neuropsychol..

[38] Ramirez-Moreno JM, Munoz-Vega P, Alberca SB, Peral-Pacheco D (2019). Health-related quality of life and fatigue after transient ischemic attack and minor stroke. J. Stroke Cerebrovasc. Dis..

[39] Sherer M (2020). Conceptual structure of health-related quality of life for persons with traumatic brain injury: Confirmatory factor analysis of the TBI-QOL. Arch. Phys. Med. Rehabil..

[40] Hansson E, Hansson T, Jonsson R (2006). Predictors for work ability and disability in men and women with low-back or neck problems. Eur. Spine J..

[41] Flensner G, Ek AC, Soderhamn O (2005). Reliability and validity of the Swedish version of the Fatigue Impact Scale (FIS). Scand. J. Occup. Ther..

[42] Crichton SL, Bray BD, McKevitt C, Rudd AG, Wolfe CD (2016). Patient outcomes up to 15 years after stroke: Survival, disability, quality of life, cognition and mental health. J. Neurol. Neurosurg. Psychiatry.

[43] Aali G, Drummond A, das Nair R, Shokraneh F (2020). Post-stroke fatigue: A scoping review. F1000Res.

[44] Moller MC, Nordin LE, Bartfai A, Julin P, Li TQ (2017). Fatigue and cognitive fatigability in mild traumatic brain injury are correlated with altered neural activity during vigilance test performance. Front. Neurol..

[45] Wolters Gregorio G (2015). Associations between executive functioning, coping, and psychosocial functioning after acquired brain injury. Br. J. Clin. Psychol..

[46] Hackett ML, Kohler S, O'Brien JT, Mead GE (2014). Neuropsychiatric outcomes of stroke. Lancet Neurol..

[47] Akerlund E, Esbjornsson E, Sunnerhagen KS, Bjorkdahl A (2013). Can computerized working memory training improve impaired working memory, cognition and psychological health?. Brain Inj..

[48] Bjorkdahl A, Akerlund E, Svensson S, Esbjornsson E (2013). A randomized study of computerized working memory training and effects on functioning in everyday life for patients with brain injury. Brain Inj..

[49] Wechsler D (2003). WAIS-III Manual.

[50] Lezak Muriel Deutsch HDB, Bigler Erin D, Tranel D (2012). Neuropsychological Assessment.

[51] Fisk JD (1994). Measuring the functional impact of fatigue: Initial validation of the fatigue impact scale. Clin. Infect. Dis..

[52] Brooks R (1996). EuroQol: The current state of play. Health Policy.

[53] Burstrom K (2014). Swedish experience-based value sets for EQ-5D health states. Qual. Life Res. Int. J. Qual. Life Asp. Treat. Care Rehabil..

[54] Wilson BA, Alderman N, Burgess PW, Ernslie H, Evans JJ (1999). Behavioural Assessment of the Dysexecutive Syndrome (BADS).

[55] Pedrero-Perez EJ (2011). Prefrontal symptoms assessment: Psychometric properties and normative data of the Dysexecutive Questionnaire (DEX) in a sample from the Spanish population. Rev. Neurol..

[56] Zigmond AS, Snaith RP (1983). The hospital anxiety and depression scale. Acta Psychiatr. Scand..

[57] Snaith RP (2003). The hospital anxiety and depression scale. Health Qual. Life Outcomes.

[58] Wechsler D (1997). WAIS III, WMS III Technical Manual.

[59] Fukuda K, Awh E, Vogel EK (2010). Discrete capacity limits in visual working memory. Curr. Opin. Neurobiol..

[60] Domholdt E (2000). Physical Therapy Research: Principles and Applications.

